# Maternal Co-ordinate Gene Regulation and Axis Polarity in the Scuttle Fly *Megaselia abdita*

**DOI:** 10.1371/journal.pgen.1005042

**Published:** 2015-03-10

**Authors:** Karl R. Wotton, Eva Jiménez-Guri, Johannes Jaeger

**Affiliations:** 1 EMBL/CRG Research Unit in Systems Biology, Centre for Genomic Regulation (CRG), Barcelona, Spain; 2 Universitat Pompeu Fabra (UPF), Barcelona, Spain; New York University, UNITED STATES

## Abstract

Axis specification and segment determination in dipteran insects are an excellent model system for comparative analyses of gene network evolution. Antero-posterior polarity of the embryo is established through systems of maternal morphogen gradients. In *Drosophila melanogaster*, the anterior system acts through opposing gradients of Bicoid (Bcd) and Caudal (Cad), while the posterior system involves Nanos (Nos) and Hunchback (Hb) protein. These systems act redundantly. Both Bcd and Hb need to be eliminated to cause a complete loss of polarity resulting in mirror-duplicated abdomens, so-called bicaudal phenotypes. In contrast, knock-down of *bcd* alone is sufficient to induce double abdomens in non-drosophilid cyclorrhaphan dipterans such as the hoverfly *Episyrphus balteatus* or the scuttle fly *Megaselia abdita*. We investigate conserved and divergent aspects of axis specification in the cyclorrhaphan lineage through a detailed study of the establishment and regulatory effect of maternal gradients in *M*. *abdita*. Our results show that the function of the anterior maternal system is highly conserved in this species, despite the loss of maternal *cad* expression. In contrast, *hb* does not activate gap genes in this species. The absence of this activatory role provides a precise genetic explanation for the loss of polarity upon *bcd* knock-down in *M*. *abdita*, and suggests a general scenario in which the posterior maternal system is increasingly replaced by the anterior one during the evolution of the cyclorrhaphan dipteran lineage.

## Author Summary

The basic head-to-tail polarity of an animal is established very early in development. In dipteran insects (flies, midges, and mosquitoes), polarity is established with the help of so-called morphogen gradients. Morphogens are regulatory proteins that are distributed as a concentration gradient, often involving diffusion from a localised source. This graded distribution then leads to the concentration-dependent activation of different target genes along the embryo’s axis. We examine this process, which differs to a surprising extent between dipteran species, in the scuttle fly *Megaselia abdita*, and compare our results to the model organism *Drosophila melanogaster*. In this way, we not only gain insights into how the mechanisms that establish polarity function differently in different species, but also how the system has evolved since these two flies shared a common ancestor. Specifically, we pin down the main difference between *Drosophila* and *Megaselia* in the altered function of the maternal Hunchback morphogen gradient, which activates target genes in the former, but not the latter species, where it has been completely replaced by the Bicoid morphogen during evolution.

## Introduction

Axis formation and segment determination in the vinegar fly *Drosophila melanogaster* are among the most thoroughly studied developmental processes today [1–5]. They offer an ideal starting point for the comparative study of development and the evolution of pattern-forming gene regulatory networks. Axis formation in flies is based on the graded distribution of morphogens established through a number of different maternal regulatory systems. In this study, we will be focusing on two of those in particular: the anterior and posterior systems [4].

In *D*. *melanogaster*, maternal protein gradients are either formed by localisation of mRNA at the anterior or posterior pole of the embryo, or by regionally specific translational repression of ubiquitous maternal transcripts [4,5]. The anterior system centres around the anterior determinant Bicoid (Bcd). *bcd* mRNA is localised to the anterior pole of the embryo and an antero-posterior (A–P) protein gradient forms through diffusion from that source [6–8]. Bcd regulates the translation of uniformly distributed maternal mRNA of *caudal (cad)* [9,10], which leads to a graded distribution of Cad protein with high concentration levels in the posterior [6,9,11–14]. In addition, Bcd acts as a concentration-dependent transcriptional regulator of zygotically expressed segmentation genes—such as gap or pair-rule genes [6,15–20]. In the case of the posterior maternal system, *nanos (nos)* mRNA is localised in the posterior pole region forming the source of the Nos protein gradient [21–23]. Unlike Bcd, Nos is not a transcriptional regulator: its only role is to translationally regulate ubiquitous maternal *hunchback (hb)* mRNA, leading to an anterior gradient of maternal Hb protein [24–26].

**Fig 1:**
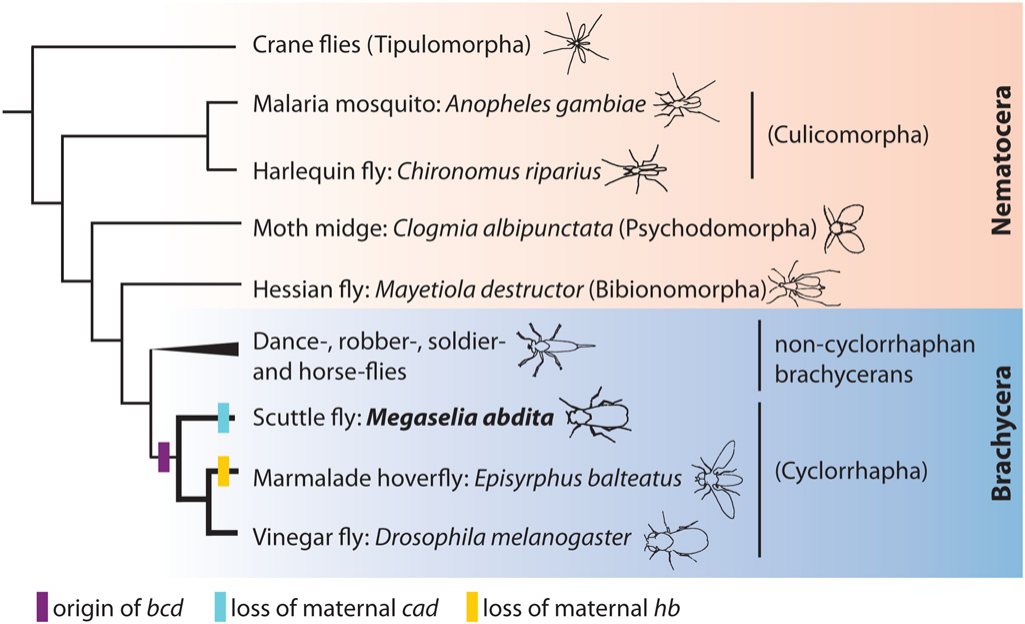
Phylogenetic position of *M*. *abdita*. This figure shows a simplified phylogenetic tree of the order Diptera (based on [45,46]), subdivided into the monophyletic suborder Brachycera (blue background) and a paraphyletic assemblage of basally branching lineages, the Nematocera (pink background). Infraorder names are shown in parentheses. Non-cyclorraphan brachycerans include the infraorders Asiloidea, Stratiomyomorpha, Tabanomorpha (robber, soldier, and horse flies), and the likely sister group of the Cyclorrhapha, the Empidoidea (dance flies). Gain and loss of maternal factors within the Cyclorrhapha are indicated by coloured bars.

Evidence for the presence of localised determinants in dipterans goes back to early studies that utilised UV irradiation or RNAse treatment on embryos of chironomid midges (Fig. 1, Nematocera: Culicomorpha). These experiments produced mirror-duplicated abdomens, so-called bicaudal phenotypes, in which anterior structures are missing and replaced by duplicated organs usually found in the posterior [27–29]. The observed effects were attributed to the destruction of an anteriorly localised mRNA. However, the identity of the anterior determinant is still unknown in the majority of dipteran infraorders. The *bcd* gene arose through a duplication of the hox3 factor *zerknüllt (zen)* at the base of the cyclorrhapha (Fig. 1) [30–33]. While its spatial distribution and role as transcriptional regulator are highly conserved among cyclorrhaphans [30–32,34–39], it is not present in other flies.

Interestingly, anterior UV irradiation of *D*. *melanogaster* embryos—or mutations to the *bcd* gene—never produce bicaudal phenotypes [40,41]. This hints at the presence of an additional non-localised factor. This factor is *hb*, which contributes to axis specification and A–P polarity in *D*. *melanogaster*. The ubiquitous distribution of its maternal mRNA may explain why it is resistant to localised UV irradiation. This interpretation is consistent with the fact that only embryos lacking both *bcd* and *hb* show bicaudal phenotypes in this species [24,42,43].

While the roles of *bcd* and *hb* in axis specification appear to be somewhat redundant in *D*. *melanogaster*, the situation is different in other cyclorrhaphan flies. The hoverfly *Episyrphus balteatus*, for example, has secondarily lost maternal *hb* expression (Fig. 1) [38,44]. Consequently, knock-down of *bcd* by RNA interference (RNAi) leads to bicaudal phenotypes in this species [38].

In this paper, we study axis specification and maternal regulation of segmentation genes in another non-drosophilid cyclorrhaphan species, the scuttle fly *Megaselia abdita* (Fig. 1). *M*. *abdita* belongs to the most basally branching cyclorrhaphan lineage, the Phoridae [45,46]. While maternal *cad* expression has been lost in this species (Fig. 1) [47], *hb* retains its maternal contribution [31]. In light of this, it is surprising that knock-down of *bcd* does lead to bicaudal phenotypes. We investigate the regulatory causes of this phenomenon through a detailed study of the establishment and regulatory role of maternal gradients in *M*. *abdtia*. Our results reveal that the anterior and posterior systems are much less redundant compared to *D*. *melanogaster*. In particular, the difference between the two species can be explained by the loss of gap gene activation through maternal *hb* in *M*. *abdita*. Our results indicate that the role of the posterior system in axis specification has been lost in *E*. *balteatus* and *M*. *abdita*, while it still retains some of its ancestral functionality in *D*. *melanogaster*. In this general scenario, the anterior system is gradually replacing the posterior one during the evolution of the cyclorrhaphan flies.

## Results and Discussion

### The posterior system establishes a maternal Hb gradient in *M*. *abdita*

**Fig 2:**
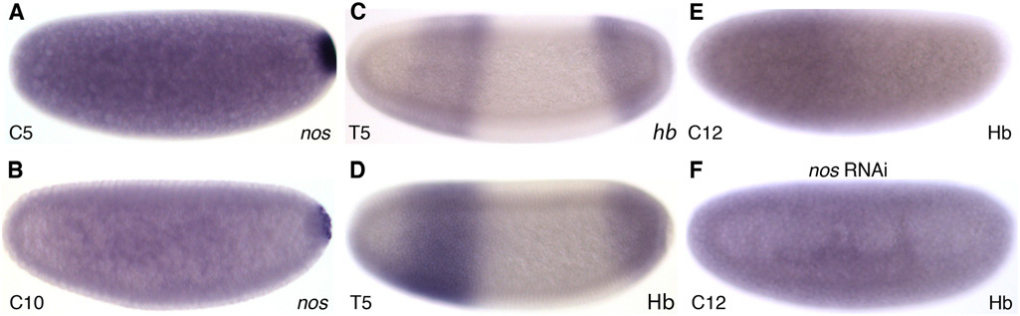
The posterior maternal system and formation of the maternal Hb gradient in *M*. *abdita*. (A,B) Expression of *nos* mRNA in wild-type embryos at C5 (A) and C10 (B). (C,D) Hb protein expression (D) resembles the mRNA pattern (C) at late blastoderm stage (C14A-T5). (E) An anterior gradient of Hb protein can be observed during cleavage and early blastoderm stages (shown for C12). (F) This gradient is abolished in embryos treated with *nos* RNAi. Images show colorimetric *in situ* hybridisation (A–C) or antibody staining (D–F). Embryo images show lateral views: anterior is to the left, dorsal is up. Time classes according to [56] are indicated at bottom left in each panel.

The posterior maternal system is based on maternal gradients of Nos and Hb protein. In *M*. *abdita*, *nos* mRNA is localised posteriorly during early cleavage stages (Fig. 2A) becoming restricted to the pole cells by C10 (Fig. 2B) as in *D*. *melanogaster*. Previous reports have documented ubiquitous maternal *hb* mRNA [31] as well as conserved zygotic *hb* expression in an anterior and a posterior domain [48]. Antibody stainings reveal a distribution of Hb protein very similar to the zygotic mRNA pattern during the late blastoderm (Fig. 2C,D). Furthermore, an anterior Hb protein gradient is present at cleavage and early blastoderm stages (Fig. 2E). In order to investigate the role of the posterior system in the formation of this gradient, we treated *M*. *abdita* embryos with *nos* RNAi. These embryos show no effect on *hb* mRNA, while ectopic Hb protein is present in the posterior of the embryo (effect detectable in 15 out of 16 RNAi-treated embryos; Fig. 2F). We conclude that the maternal Hb gradient is set up through translational repression by Nos in *M*. *abdita* as in *D*. *melanogaster*.

### Regulation of *cad* in *M*. *abdita*

The anterior maternal system of *M*. *abdita* is less conserved than the posterior one. Unlike *D*. *melanogaster* [9,10] and *E*. *balteatus* [44], *M*. *abdita* lacks maternal *cad* transcripts [47] and consequently maternal Cad protein. Zygotic expression of *cad*, on the other hand, is qualitatively similar in *D*. *melanogaster*, *E*. *balteatus*, and *M*. *abdita* [9–12,14,37,42,47–49]. The only notable difference is that abdominal *cad* expression reaches further anterior in the latter two species compared to *Drosophila* [44,48].

**Fig 3:**
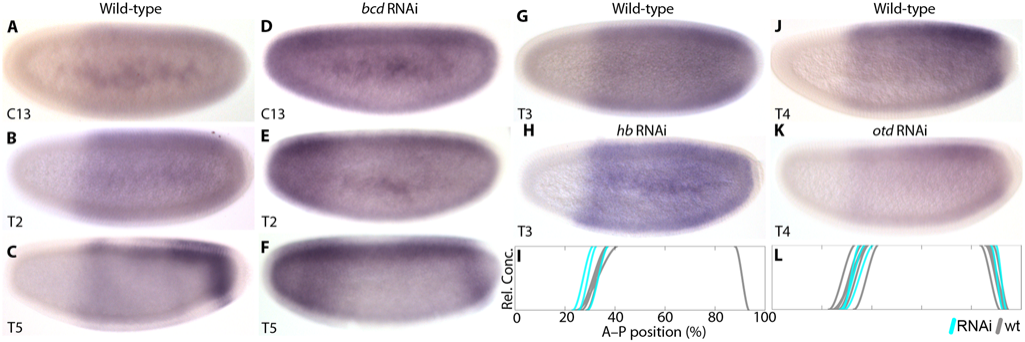
Zygotic *cad* mRNA expression in wild-type and RNAi-treated embryos. (A–F) The effect of *bcd* knock-down on *cad* expression. (A–C) show wild-type and (D–F) *bcd* RNAi-treated embryos. (G–I) *hb* knock-down. (G) shows wild-type, (H) *hb* RNAi-treated embryos. (I) compares the position of anterior *cad* expression boundaries between wild-type (wt; grey) and *hb* knock-down embryos (cyan). (J–L) *otd* knock-down. (J) shows wild-type, (K) *otd* RNAi-treated embryos. (L) compares the position of anterior *cad* expression boundaries between wild-type (wt; grey) and *otd* knock-down embryos (cyan). Embryo images show lateral views: anterior is to the left, dorsal is up. Time classes according to [56] are indicated at bottom left in each panel. Summary graphs in (I) and (L): horizontal axes represent % A–P position (with 0% at the anterior pole); vertical axes indicate relative mRNA concentration in arbitrary units (au).

In order to test how zygotic *cad* expression is regulated in *M*. *abdita*, we knocked down *bcd*, *hb*, and the head gap gene *orthodenticle (otd)*. In *bcd* RNAi-treated embryos, we observe a derepression of *cad* transcripts in the anterior (38/48; Fig. 3A–F). At cleavage cycle 13 (C13), *cad* expression appears uniform throughout the embryo (Fig. 1D). During early C14A (time class 2, T2), *cad* becomes expressed at higher levels in the anterior than in the posterior (Fig. 3E). This effect is specifically confined to the region that is free of *cad* expression in wild-type embryos (compare to Fig. 3B). At later stages, an ectopic domain resembling the posterior *cad* stripe forms in the anterior (Fig. 3F). Similar ectopic *cad* stripes have been observed in the anterior of *D*. *melanogaster bcd* mutants [14], *cad* reporter assays in *D*. *melanogaster* [47], and *E*. *balteatus* embryos treated with *bcd* RNAi [38].

In *hb* knock-down embryos, we observe a small anterior expansion of *cad* expression in a minority of specimens (4/13; Fig. 3G–I; S1 File). Anterior derepression is much more subtle in this case than in *bcd* knock-downs (Fig. 3D–F). This effect is similar to *hb* mutants of *D*. *melanogaster* [42].

Given the difference between *bcd* and *hb* knock-downs, we investigated potential additional contributions by *otd*, a factor known to act as a transcriptional repressor of *cad* in the jewel wasp *Nasonia vitripennis* [50]. *otd* expression is lost in *bcd* RNAi-treated embryos (12/16; S1 Fig). However, expression of *cad* appears normal in embryos treated with *otd* RNAi (25/25; Fig. 3J–L; S1 File). This indicates that *otd* is not involved in *cad* regulation, consistent with results from *D*. *melanogaster* [50] and *E*. *balteatus* [44].

In summary, anterior repression of *cad* in *D*. *melanogaster* is due mainly to a combination of translational repression by Bcd—acting on ubiquitous maternal *cad* mRNA—and transcriptional repression by *hb—*acting on the zygotic abdominal *cad* domain [14,42]. Transcriptional regulation of *cad* by Bcd plays a minor role, if any [47]. In contrast, repression of *cad* by Bcd occurs predominantly at the transcriptional rather than the translational level in *M*. *abdita*, similar to *E*. *balteatus* [38]. Our evidence does not conclusively establish whether this interaction is direct. However, we have shown that potential intermediate factors such as Otd and Hb are not involved in *cad* regulation, or show regulatory effects that are far too subtle to account for anterior repression in *M*. *abdita*.

### The role of *bcd* in *M*. *abdita*

**Fig 4:**
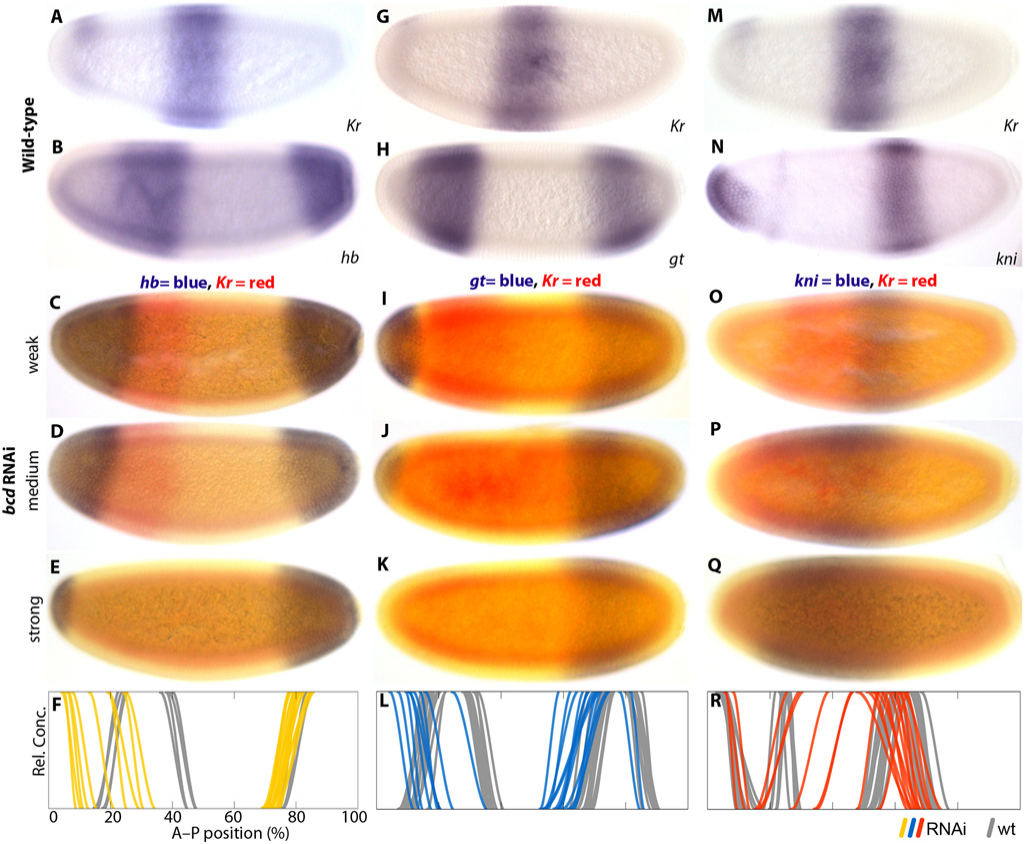
Gap gene expression in response to *bcd* RNAi knock-down. Columns show wild-type (A,B,G,H,M,N) or *bcd* RNAi embryos (C–E,I–K,O–Q) single- or double-stained for *Kr* (A,C–E,G,I–K,M,O–Q) along with *hb* (B–F), *gt* (H–L), *and kni* (N–R) as indicated. (F,L,R) Summary graphs comparing wild-type boundary positions (wt; grey lines) to boundary positions affected by RNAi (coloured lines). *Kr* is stained in red (C–E, I–K, O–Q) or blue (A,G,M). Other stains as indicated. All embryos are at time class T4 (*hb and kni* columns) or T3 (*gt* column). Embryo images show lateral views: anterior is to the left, dorsal is up.

**Fig 5:**
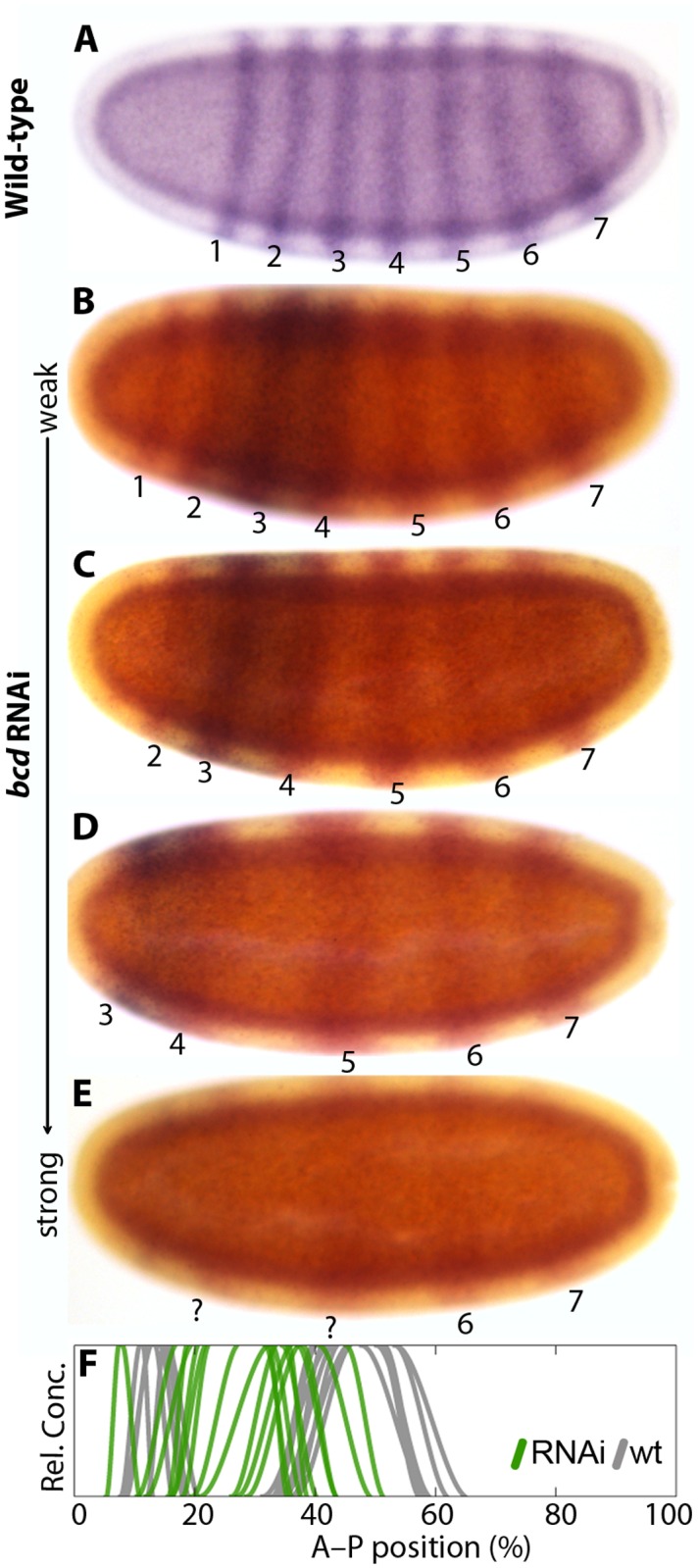
*eve* and *Kr* expression in response to *bcd* knock-down. (A) Wild-type *eve* expression. (B–E) Weak to strong phenotypes in embryos treated with *bcd* RNAi: embryos were double-stained for *eve* (red) and *Kr* (blue). Numbers indicate *eve* stripes 1–7. (F) Summary graph comparing wild-type *Kr* boundary positions (wt; grey lines) to boundary positions affected by RNAi (green lines). All embryos are at time class T5. Embryo images show lateral views: anterior is to the left, dorsal is up.

Previous work has shown that *bcd* mRNA is localised anteriorly in *M*. *abdita* [30–32,48], and that it regulates *hb* transcription through the P2 promoter [31,37]. To assess the effect of *bcd* on gap gene regulation and embryo polarity in general, we characterised expression patterns of the trunk gap genes *hb*, *giant (gt)*, *knirps (kni)*, *Krüppel (Kr)*, and the pair-rule gene *even-skipped (eve)* in *M*. *abdita* embryos treated with *bcd* RNAi. We used single- and double-stained embryos to assess severity of the knock-down and spatial registration of expression patterns—between gap domains (Fig. 4) as well as between *Kr* and the pair-rule gene *eve* (Fig. 5). We take advantage of the variable knock-down efficiency in RNAi experiments, which acts similar to an allelic series in classical genetics, to measure the sensitivity of specific gap domain boundaries towards decreasing levels of Bcd. In general, we find that all of these boundaries are highly sensitive to changes in Bcd concentration (Figs. 4 and 5; see also S1 File).

Wild-type embryos of *M*. *abdita* show a broad, *bcd-*dependent, anterior domain of zygotic *hb* expression, which gradually retracts from the pole (Fig. 4B) [31,37]. The posterior boundary of this domain shifts in anterior direction over time [48], unlike its equivalent in *D*. *melanogaster*. In embryos treated with *bcd* RNAi, we observe an anterior cap of *hb* expression which never retracts from the pole (35/42; Fig. 4C–F; S1 File). It reduces in size with the severity of the *bcd* knock-down (Fig. 4C–E) indicating dependence on Bcd concentration. Similar anterior domains have been observed in embryos derived from *bcd* mutant mothers in *D*. *melanogaster* [51] and in *bcd* RNAi-treated embryos of *E*. *balteatus* [38]. In both of these cases, the anterior cap of *hb* expression has been interpreted as an anterior mirror duplication of the posterior *hb* domain [38,51]. The posterior *hb* domain is also conserved in *M*. *abdita* (Fig. 4B) [48]. It exhibits a slight anterior expansion in some embryos treated with *bcd* RNAi (Fig. 4C–F; S1 File). In contrast, the posterior *hb* domain remains unaffected in *D*. *melanogaster* embryos lacking *bcd* [51].

Wild-type embryos of *M*. *abdita* have a broad anterior domain of *gt*, with a stationary posterior boundary, plus a posterior domain that shifts anteriorly over time (Fig. 4H) [48]. In embryos treated with *bcd* RNAi, we observe either loss (10/18) or strong reduction (8/18) of the anterior *gt* domain at early stages (before T3), while most embryos exhibit expression in a small anterior cap at later time points (14/15; Fig. 4I–L; S1 File). This anterior cap retracts from the pole around T8 (1/1). As for *hb*, the extent of anterior *gt* expression decreases with increasing strength of the knock-down effect (Fig. 4I–K). We interpret these observations as follows: delay and reduction of anterior *gt* expression are due to a lack of activation by Bcd, while the late anterior cap domain may be induced by ectopically expressed Cad (see Fig. 3E, F). The effect of *bcd* knock-down on the posterior *gt* domain is more modest. This domain is always present in *bcd* RNAi embryos but exhibits some anterior displacement of both its boundaries (Fig. 4I–L; S1 File). *D*. *melanogaster* embryos from *bcd* mutant mothers show a similar anterior displacement of the posterior *gt* domain, but no expression of *gt* in the anterior [52,53]. In contrast, *E*. *balteatus* embryos treated with *bcd* RNAi show broad derepression of *gt*, whose expression is only excluded from the anterior and posterior tip of the embryo [38].

In wild-type embryos of *M*. *abdita*, *kni* is expressed in an L-shaped anterior head domain, plus an abdominal domain that shifts to the anterior over time (Fig. 4N) [48]. In embryos treated with *bcd* RNAi, the head domain disappears, while the abdominal domain of *kni* expands and becomes displaced towards the anterior (38/38; Fig. 4O–R; S1 File). As in the case of *hb* and *gt*, the amount of expansion depends on the severity of the knock-down. This is qualitatively similar to embryos derived from *bcd* mutant mothers in *D*. *melanogaster*, but the effect is more severe in *M*. *abdita* and resembles *kni* expression in *bcd* mutants which are also heterozygous for maternal *hb* [24]. The effect of Bcd on *kni* is even more pronounced in *E*. *balteatus* where *kni* becomes drastically derepressed—showing ubiquitous expression in extreme cases—in embryos treated with *bcd* RNAi [38].

Wild-type *M*. *abdita* embryos have a central *Kr* domain, which is wider than its equivalent in *D*. *melanogaster* (Fig. 4A, G, M) [48]. As is the case for other gap domains, it shifts anteriorly and contracts over time. In embryos treated with *bcd* RNAi, the central domain of *Kr* expands towards the anterior (94/116; Fig. 4C–E, I–K, O–Q; Fig. 5B–F; S1 File). Yet again, the extent of the expansion is correlated with the strength of the knock-down. In the strongest cases, *Kr* expression is entirely missing (22/116; Fig. 4E, K, Q). A similar expansion of the central *Kr* domain has been observed in embryos from *bcd* mutant mothers in *D*. *melanogaster* [24]. However, these embryos never show a complete lack of *Kr* expression; it is only abolished by the additional removal of maternal *hb* [24,54]. Knock-down of *bcd* in *E*. *balteatus*, which lacks maternal *hb* expression altogether, leads to a complete absence of *Kr* expression in all RNAi-treated embryos [38].

In summary, our results suggest that Bcd is a concentration-dependent transcriptional regulator of gap genes in *M*. *abdita*. The observed effects of Bcd on gap gene expression are more severe than in *D*. *melanogaster* (resembling gap gene patterns in mutants affecting both *bcd* and *hb*), but milder than in *E*. *balteatus*.

### *Kr* expression and polarity reversal in *M*. *abdita*

*M*. *abdita* embryos treated with *bcd* RNAi can exhibit a bicaudal phenotype with complete axis polarity reversal and mirror-duplicated posterior structures in the anterior [31]. These severe knock-down phenotypes have their plane of symmetry at abdominal segment 5 (A5), and express four *eve* stripes—the two anterior ones probably being mirror-duplicated stripes 6 and 7 [31]. Such polarity reversal is never observed in embryos derived from *bcd* mutant mothers in *D*. *melanogaster* [41], only in embryos that lack both *bcd* and maternal *hb* [24,43,54]. While the former still have a residual *Kr* domain, the latter lack *Kr* expression completely. Polarity reversal is also observed in *E*. *balteatus* embryos treated with *bcd* RNAi, which show no *Kr* expression at all [38].

We tested the relationship between the bicaudal phenotype and the presence or absence of *Kr* by co-staining *bcd* knock-down embryos for both *eve* and *Kr* (Fig. 5). The pair-rule gene *eve* is expressed in seven stripes in wild-type *M*. *abdita* embryos (Fig. 5A) [44,55,56]. Weak *bcd* knock-down phenotypes show a full complement of seven *eve* stripes that are displaced towards the anterior, with a correspondingly mild anterior displacement of *Kr* (Fig. 5B; compare to Fig. 4C, I, O). Increasing severity of the knock-down results in the progressive loss of anterior *eve* stripes and more pronounced anterior displacement of the central *Kr* domain (Fig. 5C–E; compare to Fig. 4D, J, P). In the strongest cases, we detect four *eve* stripes only (as in [31]), and no or very little *Kr* expression (Fig. 5F; compare to Fig. 4E, K, Q). This suggests that the absence of *Kr* expression is correlated with polarity reversal in *bcd* knock-down embryos.

### Differing roles of maternal *hb* in *M*. *abdita* and *D*. *melanogaster*

Why does lack of Bcd induce a bicaudal phenotype in *M*. *abdita* if it has a maternal Hb gradient very similar to *D*. *melanogaster*? To answer this question, we compared the role of maternal Hb in gap gene regulation in both species.

We have previously characterised the effect of Hb on *Kr*, *kni*, and *gt* in *M*. *abdita* [48]. Expression of *kni* and *gt* in embryos treated with *hb* RNAi is very similar to the corresponding patterns in *hb* mutants of *D*. *melanogaster*. In contrast, the effect of Hb on *Kr* differs between the two species: both show an anterior expansion of the central *Kr* domain (24 out of 53 RNAi-treated embryos in *M*. *abdita*), but only *D*. *melanogaster* embryos lacking maternal Hb exhibit a decrease in *Kr* expression levels [24,54]. We never observe such down-regulation in *M*. *abdita* embryos treated with *hb* RNAi (S2 Fig) [48]. Together with the absence of *Kr* expression in strong *bcd* knock-down phenotypes (Fig. 4E, K, Q, Fig. 5E), this indicates that Hb is unable to activate *Kr* in *M*. *abdita*.

In contrast, several authors have interpreted the reduced levels of *Kr* expression in *hb* mutants as evidence for activation of *Kr* by Hb in *D*. *melanogaster* [24,42]. However, it has never been shown whether this activating effect is direct or indirect—via repression of the repressor Kni by Hb (see [5], for a detailed discussion). To distinguish between these two possibilities, it is necessary to suppress *kni* in a background lacking maternal and zygotic *hb*. Direct activation is supported if levels of *Kr* expression remain low in embryos lacking both *hb* and *kni*, while an indirect effect via *kni* is supported if *Kr* levels are restored in these embryos compared to *hb* mutants alone. Unfortunately, it is not straightforward to create such double mutants, since both *hb* and *kni* are located on the same chromosome in the *D*. *melanogaster* genome, and germ line clones must be induced to eliminate both maternal and zygotic activities of *hb*. This may be the reason why this experiment has never been carried out. To overcome this challenge, we used RNAi-mediated double knock-down of *hb* and *kni*, and knock-down of *hb* in a *kni* mutant background.

**Fig 6:**
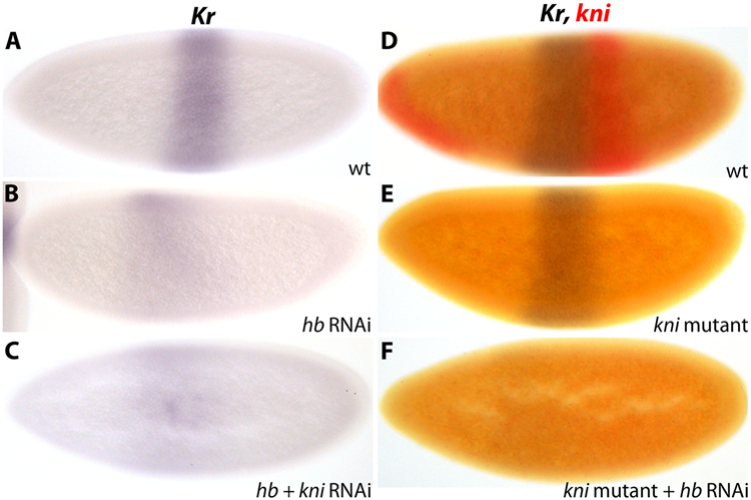
*Kr* is activated by maternal Hb in *D*. *melanogaster*. (A–C) *Kr* expression in wild-type embryos (wt; A) versus embryos treated with *hb* RNAi (B) and *hb/kni* double RNAi (C). (D–F) *Kr* (blue) and *kni* (red) expression in wild-type (wt; D) versus *kni* mutants (E) and *kni* mutants treated with *hb* RNAi (F). See text for details. All embryos are at time class T4. Embryo images show lateral views: anterior is to the left, dorsal is up.

In *D*. *melanogaster hb* knock-down embryos, we observe anterior expansion and strong down-regulation of *Kr* (5/9; Fig. 6A, B; S2 File), as well as considerable anterior displacement of *kni* (3/5; S3 Fig; S2 File). These patterns correspond precisely to *Kr* and *kni* expression in embryos mutant for both maternal and zygotic *hb* [24]. Similarly, *kni* knock-down embryos show a *Kr* pattern which is identical to that observed in *kni* null mutants: we observe no posterior expansion of *Kr* (Fig. 6E; S2 File), in accordance with a recent quantitative study [57], but in disagreement with earlier qualitative reports [58–60]. These results indicate that our early embryonic RNAi knock-downs mimic strong null mutant phenotypes.

In *D*. *melanogaster hb/kni* double knock-down embryos, we observe an anterior expansion of *Kr*, but no restoration of expression levels (12/18; Fig. 6C; S2 File). We confirm this result in *kni* mutant embryos treated with *hb* RNAi, which exhibit an identical anterior expansion of *Kr* and no restoration of expression levels (12/14; Fig. 6F; S2 File). Taken together, these results demonstrate that *kni* is not responsible for *Kr* down-regulation in *D*. *melanogaster* embryos lacking maternal and zygotic Hb. Therefore, activation of *Kr* by Hb is direct in this species. In contrast, this activatory role is absent in *M*. *abdita* where Hb acts as a repressor only, which leads to a lack of *Kr* expression and mirror symmetrical expression of the remaining gap genes in *bcd* knock-down embryos (see also Conclusions).

### The role of zygotic *cad* in *M*. *abdita*

**Fig 7:**
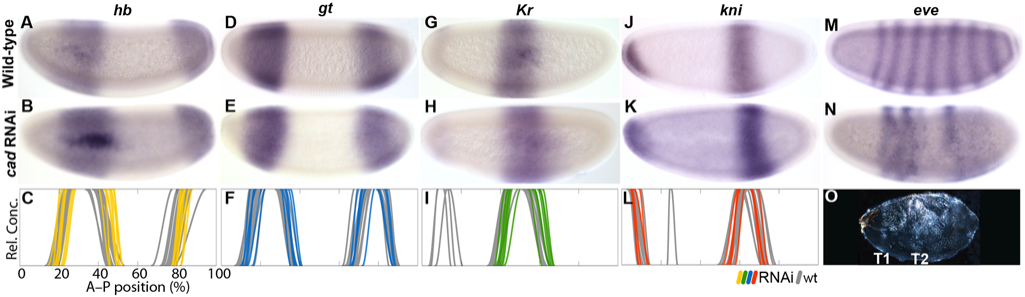
RNAi knock-down of *M*. *abdita cad*. Columns show the expression of *hb* (A–C; yellow), *gt* (D–F; blue), *Kr* (G–I; green), *kni* (J–L; red), and *eve* (M,N) in wild-type embryos (top row: A,D,G,J,M), in embryos treated with *cad* RNAi (middle row: B,E,H,K,N), and as summary graphs comparing wild-type (wt) boundary positions (grey) to those affected by RNAi (coloured lines) (bottom row: C,F,I,L). (O) Cuticle phenotype of an embryo treated with *cad* RNAi. All embryos are at time class T4. Embryo images show lateral views: anterior is to the left, dorsal is up. Graphs: horizontal axes indicate % A–P position (where 0% is the anterior pole); vertical axes represent relative mRNA concentration in arbitrary units.

In *D*. *melanogaster*, maternal and zygotic Cad contribute to the activation of posterior gap domains [13,42] and—at least partially independently of gap gene regulation—activate posterior stripes of pair rule gene expression [11,50,61–64]. To investigate the exclusively zygotic contribution of Cad to gap and pair-rule gene expression in *M*. *abdita*, we characterised the expression patterns of *hb*, *gt*, *Kr*, *kni*, and *eve* (Fig. 7A–L; S1 File) as well as the cuticle phenotype (Fig. 7O) of embryos treated with *cad* RNAi. The *cad* knock-down phenotype of *M*. *abita* exhibits deletions of all segments posterior of T3, and T3 itself is also disrupted in some embryos (Fig. 7O). This phenotype is more similar to *D*. *melanogaster* than to *E*. *balteatus*. Embryos of the latter treated with *cad* RNAi exhibit a strongly reduced cephalopharyngeal skeleton, in addition to an almost complete loss of abdomen and thorax [44]. In contrast, *D*. *melanogaster* embryos mutant for both maternal and zygotic *cad* have an intact head and thorax and, although there is extensive loss of abdominal segments, often even retain some abdominal structures [11]. The fact that the *M*. *abdita* phenotype is stronger than that of *D*. *melanogaster* suggests that *cad* still plays an essential role in posterior segmentation in this species despite the loss of its maternal contribution.

In light of this, it is surprising that knock-down of *cad* in *M*. *abdita* does not have a strong effect on gap gene expression. The only clearly detectable defect is a slightly reduced posterior *hb* domain (7/16; Fig. 7A–C). All other domains of *hb*, *gt*, *Kr*, and *kni* seem unaffected (Fig. 7A–L; see also S1 File). Expression levels of *Kr*, *kni*, and *gt* appear similar to wild-type, although we cannot completely rule out a marginal decrease due to lack of sensitivity of our enzymatic detection method. This stands in contrast to *D*. *melanogaster*, where expression levels in the abdominal domain of *kni* and the posterior domain of *gt* are reduced in mutants lacking both zygotic and maternal *cad* (while *hb* and *Kr* are expressed as in wild-type) [13,42,50]. In *E*. *balteatus cad* knock-down embryos, anterior *hb* and *Kr* are normal, while the posterior *kni*, *gt*, and *hb* domains are absent or severely reduced [38,44].

**Fig 8:**
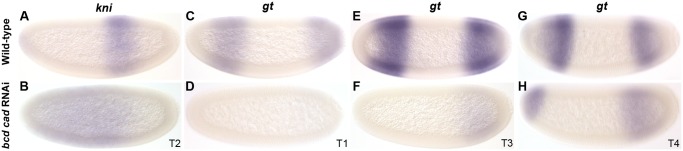
RNAi double knock-down of *M*. *abdita bcd* and *cad*. Columns show the expression of *kni* (A, B), and *gt* (C–H) in wild-type embryos (top row: A, C, E, G), and in embryos treated simultaneously with *bcd* and *cad* RNAi (bottom row: B, D, F, H). Embryo time classes as indicated at bottom right of each column. Embryo images show lateral views: anterior is to the left, dorsal is up.

To test if activation of gap genes by Cad is present but redundant with the complementary contribution by Bcd, we characterised the expression of *kni* and *gt* in embryos treated with RNAi against both *bcd* and *cad* (Fig. 8; see also S1 File). We observe a large anterior displacement in the position of the abdominal *kni* domain (36/36), as is seen in *bcd* RNAi-treated embryos. This was associated with a strong reduction in expression levels, particularly before T3 (Fig. 8B; 14/15), though after this stage levels of expression begin to resemble those in the wild-type. Embryos treated with *bcd* or *cad* RNAi alone, never show such reduction (Fig. 4N–Q, Fig. 7J, K). Expression of *gt* is absent before T2 (6/10), and only becomes detectable as a weak posterior domain at later stages (Fig. 8D, F). In contrast to *bcd* RNAi-treated embryos (Fig. 4H–L), we do not observe any anterior displacement of this domain (see S1 File). In the anterior, we observe a cap of *gt* expression at the late blastoderm stage (Fig. 8H), which closely resembles the anterior cap in embryos treated with RNAi against *bcd* alone (Fig. 4I,J; S1 File). Our observations stand in contrast to those from *D*. *melanogaster* mutants lacking both maternal and zygotic *cad* and *bcd*. Such mutants show complete absence of both *kni* and *gt* expression [13].

Taken together, our results suggest that Cad contributes to early activation of both abdominal *kni* and posterior *gt* in *M*. *abdita*, in a way which is largely redundant with activation by Bcd. Surprisingly, late expression of both *kni* and *gt* in the posterior of the embryo seems to be at least partially independent of both Bcd and Cad activation. This suggests that a third, yet unknown, factor must contribute to gap gene activation in this species.

Finally, we investigated the contribution of *M*. *abdita cad* to pair-rule expression. In embryos treated with *cad* RNAi, we observe a reduction in the number of *eve* stripes: 2 out of 12 embryos showed three, 5/12 four, and 5/12 five *eve* stripes (Fig. 7M,N). Similarly, *D*. *melanogaster* embryos mutant for both maternal and zygotic *cad* have four *eve* stripes [50]. The most drastic effect of *cad* on pair-rule gene expression is observed in *E*. *balteatus*, where embryos treated with *cad* RNAi exhibit the loss of all but the first stripe of *eve* [44].

Taken together, our evidence demonstrates that zygotic *cad* still plays an important role in the determination of posterior segments of *M*. *abdita*. In contrast to *D*. *melanogaster* and *E*. *balteatus*, where eliminating *cad* has a clearly detectable effect on gap gene expression [13,42,44], it is largely redundant for gap gene activation in *M*. *abdita*. This implies that *cad* performs its pattern-forming role mainly at the level of the pair-rule genes in this species.

### Conclusions

**Fig 9:**
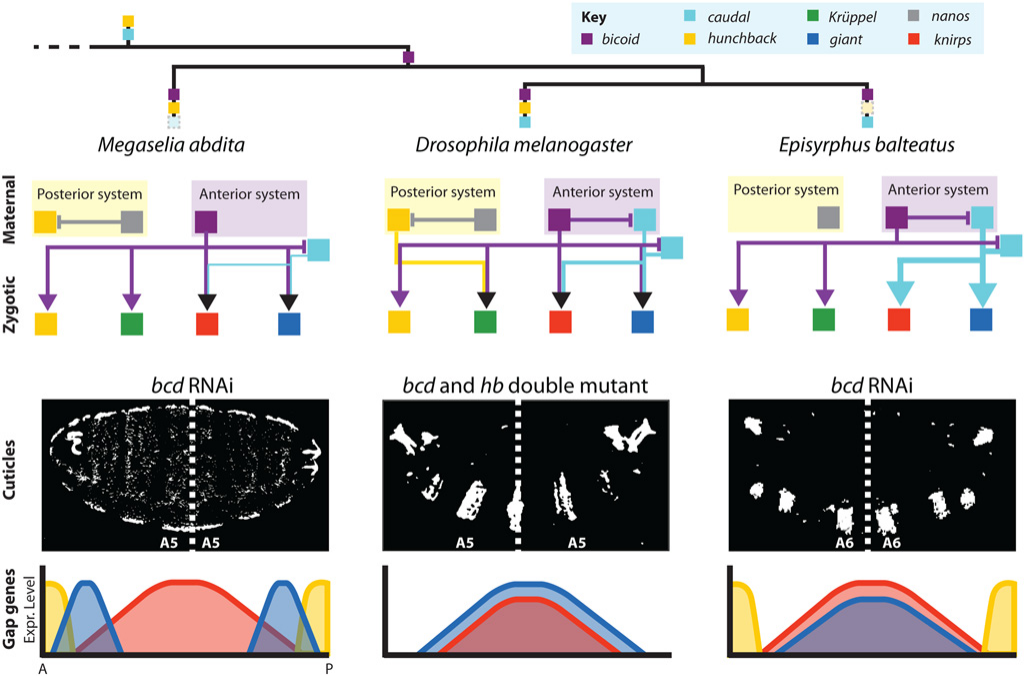
Summary of maternal gene regulatory networks and gap gene expression in embryos with bicaudal phenotypes. Top: phylogeny of species discussed in this paper indicating ancestral maternal factors (*cad* and *hb*), the origin of *bcd*, and the loss of maternal factors in different lineages (faded dashed boxes). Middle: schematic graphs showing gene regulatory networks implementing maternal contributions to gap gene activation. Translational and/or transcriptional cross-repression among maternal genes represented by T-bars, activating inputs to gap genes shown by arrows. Cross-regulation between gap genes has been analysed previously [48] and is omitted for clarity. Bottom: double-abdomen cuticles (upper row) and corresponding gap gene expression patterns (bottom row) are shown for *M*. *abdita* and *E*. *balteatus* embryos treated with *bcd* RNAi, as well as *bcd/hb* double mutants in *D*. *melanogaster*. Dashed lines indicate the plane of symmetry. *D*. *melanogaster* and *E*. *balteatus* cuticle images adapted from [24,38].

In this study, we have investigated the establishment of maternal gradients and their role in gap gene regulation in the scuttle fly *M*. *abdita*. We compare our results with the evidence from the vinegar fly *D*. *melanogaster* as well as the marmalade hoverfly *E*. *balteatus* (Fig. 9). On the one hand, we find that important aspects of maternal regulation are highly conserved among cyclorrhaphan flies. Bcd acts as a concentration-dependent transcriptional regulator, and Cad is involved in posterior patterning in all three species. On the other hand, we find a number of interesting differences between *M*. *abdita*, *E*. *balteatus*, and *D*. *melanogaster*.

The first difference concerns the regulation of *cad*. Even though maternal *cad* expression can be detected in nematocerans, and basally branching non-cyclorrhaphan brachycerans (Fig. 1), maternal expression of *cad* has been lost in *M*. *abdita* [47]. Zygotic expression of *cad* is qualitatively similar between species, but reaches further anterior in *M*. *abdita* and *E*. *balteatus* than in *D*. *melanogaster*, creating a large overlap of *cad* and *hb* in these flies. Consistent with the absence of strong repression between these two genes, *hb* only weakly affects *cad* expression in *M*. *abdita*. In contrast, *cad* is completely de-repressed anteriorly in *bcd* knock-down embryos (see Fig. 3). There is some evidence from reporter assays that Bcd may regulate *cad* transcriptionally in *D*. *melanogaster* as well [47]. The situation is much less ambiguous in the case of *E*. *balteatus*, where *cad* is strongly up-regulated in the anterior upon *bcd* RNAi knock-down [38]. This similarity between *M*. *abdita* and *E*. *balteatus* suggests that transcriptional repression of *cad* by Bcd is much more prominent in these flies compared to *D*. *melanogaster*. Whether this interaction is direct in any of the three species remains to be shown.

The second difference concerns the roles of *bcd* and *hb* in axis specification and gap gene patterning. Knock-down of *bcd* in *M*. *abdita* and *E*. *balteatus* leads to bicaudal phenotypes, as observed in *bcd/hb* double mutants but not in *bcd* mutants in *D*. *melanogaster* [24,41–43]. It is important to note that the situation in *M*. *abdita* is distinct from both *D*. *melanogaster* and *E*. *balteatus* (Fig. 9). More positional information is retained in bicaudal embryos, resulting in a more anterior (A5) plane of symmetry, compared to A6 in the latter two species [24,31,38]. This difference is also reflected at the level of gap gene expression. Severe *M*. *abdita* knock-down phenotypes for *bcd*, which lack *Kr* expression, show a sequence of *hb-gt-kni-gt-hb* domains along the antero-posterior axis (Fig. 9) (this paper and [31]). *D*. *melanogaster hb/bcd* double mutants only have overlapping central *gt* and *kni* domains (Fig. 9) [24,51,52,65]. *E*. *balteatus* knock-down embryos show an almost complete de-repression of *gt* and *kni* throughout the embryo (Fig. 9) [38].

The anterior gradient of Bcd is an evolutionary innovation of the cyclorrhaphan lineage (Fig. 1) [30–33]. The evidence suggests that it is completely sufficient for axis specification and embryo polarity in *M*. *abdita* and *E*. *balteatus*. In contrast, both maternal Bcd and Hb contribute synergistically to axis specification and gap gene patterning in *D*. *melanogaster*. While differences in the effect of Bcd between *D*. *melanogaster* and *E*. *balteatus* are easily explained by the absence of maternal *hb* in the latter [38], it is less straightforward to pinpoint the cause for polarity reversal in *bcd* knock-down embryos of *M*. *abdita*. Our evidence suggests that this difference lies in the ability of maternal Hb to activate *Kr* in *D*. *melanogaster*, but not *M*. *abdita* (see Fig. 4, and [48]). *Kr* expression in the anterior of the embryo is correlated with the maintenance of polarity in *D*. *melanogaster bcd* mutants, and weak *bcd* knock-down phenotypes in *M*. *abdita* (Figs. 4 and 5). In *D*. *melanogaster*, maternal Hb is required for *Kr* expression in the absence of Bcd [24,42], and we have shown here that this activating interaction is indeed direct and not caused by the indirect repression of the Kni repressor (Fig. 6).

It remains unclear whether activation of *Kr* by maternal Hb has been gained in *D*. *melanogaster* or lost in *M*. *abdita*. However, there is some evidence that favours the latter scenario. Maternal *hb* expression is strongly conserved across arthropods far beyond the cyclorrhaphan lineage [66–74], and *hb* is involved in axis patterning in many of the species where it has been studied [67,68,70–72,75,76]. Most interestingly in our context, Hb activates *Kr* in the flour beetle *Tribolium castaneum* [75], the honeybee *Apis mellifera* [72], the hemipteran milkweed bug *Oncopeltus fasciatus* [76], and the cricket *Gryllus bimaculatus* [69]. The fact that this activating role of *hb* is conserved, and is only present in the one cyclorrhaphan species that retains some activity of maternal Hb in axis formation, seems to suggest that it may represent the ancestral state, and that activation of *Kr* by Hb has been lost in *M*. *abdita* and *E*. *balteatus*.

We have previously demonstrated that the gap gene system of *M*. *abdita* compensates for the significant differences in the distribution of maternal factors compared to *D*. *melanogaster*, such that gap gene expression converges to equivalent patterns in both species by the onset of gastrulation [48]. Such compensatory evolution is called developmental system drift or phenogenetic drift [77–81]. At the level of the gap genes, this is achieved through quantitative changes in the strength of otherwise wholly conserved gap-gap interactions [48]. In contrast, our study shows that system drift at the level of maternal-to-gap interactions is mediated by both quantitative and qualitative differences in gene regulation. While inter-species differences in the effect of Bcd and Cad mainly consist in changes in activation strength, the activating role of Hb on *Kr* has changed in a qualitative way: while Hb activates *Kr* in *D*. *melanogaster*, this activating role is absent in both *M*. *abdita* and *E*. *balteatus* (Fig. 9).

In summary, we observe a trend towards replacing the role of maternal Hb with activity of the anterior maternal system—Bcd and Cad—in non-drosophilid cyclorrhaphan lineages through the process of developmental system drift. This is reflected by the stronger phenotypes of *bcd* and *cad* knock-downs in both *E*. *balteatus* and *M*. *abdita* compared to *D*. *melanogaster*. In this view, axis formation and gap gene patterning in *D*. *melanogaster* retains more ancestral characteristics than these early-branching non-drosophilid cyclorrhaphans. Further corroboration of these insights will have to come from functional studies of axis specification and gap gene patterning in an appropriate outgroup (Fig. 1): non-cyclorrhaphan brachycerans or emerging nematoceran model systems such as the chironomid midge *Chironomus riparius* or the moth midge *Clogmia albipunctata*.

## Methods

*M*. *abdita* fly culture, embryo collection and fixation were carried out as described in [82,83]. Enzymatic mRNA *in situ* hybridisation, image acquisition, and data processing were carried out as described in [84,85]. We use an embryonic staging scheme—homologous to the one already established for *D*. *melanogaster* [86]—which is described in detail in [56]. Embryo morphology and developmental timing are remarkably similar in both species. Embryos are classified into cleavages cycles C1–C14A according to nuclei number; C14A is further subdivided into eight time classes T1–8 based on nuclear and membrane morphology.

Polyclonal antiserum was raised against *M*. *abdita* Hb protein expressed by means of a pET-DEST42 vector (Invitrogen) containing a full length cDNA insert. Purified Hb protein dissolved in 6M urea was used to raise rat antibodies by Primm Biotech (primmbiotech.com) using standard protocols. For antibody stains, wild-type blastoderm-stage embryos were collected after 4 hrs of egg laying and stained with a colorimetric protocol adapted from the *in situ* protocol published in [85]. In brief, fixed and dehydrated embryos were re-hydrated by washing 1x5min in PBT/methanol (embryos were allowed to sink before the solution was removed), 2x in PBT, and 1x20 min in PBT. Embryos were washed 1x, then blocked with 2x30 min in western blocking reagent (Roche) in PBT followed by incubation with primary antibodies in blocking solution overnight. Unbound antibody was removed washing 3x in PBT followed by 4x15 min washes in PBT. Embryos were then re-blocked and incubated with secondary antibodies conjugated with alkaline phosphatase (Roche) at 1:3000 in blocking solution for 1 hr. Unbound antibody was removed as before. To prepare for staining, embryos were washed 2x5 min in AP buffer (100 mM NaCl, 50 mM MgCl, 100 mM Tris pH 9.5, 0.1% tween-20). Staining was carried out in the dark by the addition of AP buffer containing 0.1 mg/ml NBT and 0.05 mg/ml BCIP. Staining was stopped with 3x1 min followed by 3x10 min washes in PBT. Nuclei were counter-stained by a 10-min incubation in PBT containing 0.3 μM DAPI, followed by 3x washes and 3x10 min washes in PBT. Embryos were cleared through a series into 70% glycerol:PBS, of which 30 μl were mounted per slide. All washes were done on a nutator.

We used RNAi knock-down protocols adapted from [31,37,87]. See [48] for further details.

All expression boundaries plotted as graphs were extracted from NBT/BCIP stained embryos, except for *Kr* expression in *M*. *abdita bcd* RNAi-treated embryos, where boundaries were extracted from FastRed stains. Differences in expression levels in Fig. 6 and S2 Fig were assessed through simultaneous staining of wild-type and RNAi-treated embryos using NBT/BCIP to ensure a robust signal.

Quantified expression data for *M*. *abdita* wild-type and RNAi knock-down embryos are available online through figshare (http://dx.doi.org/10.6084/m9.figshare. 1252195; [88], and the SuperFly database (http://superfly.crg.eu; [89]). Plots of gene expression boundaries from RNAi-treated or mutant embryos can be found in S1 File (*M*. *abdita*) and S2 File (*D*. *melanogaster*).

*nos* (KP232978) was cloned from cDNA using data from our published early embryonic transcriptome (http://diptex.crg.es; MAB_comp4961) [46]. All other genes were cloned as described in [48].

Embryo collection, fixation, RNAi treatment, and *in situ* hybridisation in *D*. *melanogaster* was carried out as for *M*. *abdita* [85,87]. *D*. *melanogaster kni* mutants correspond to deletion strain 3127 (Bloomington Drosophila Stock Center) with genotype *Df(3L)ri-79c/TM3*, *Sb* ^*1*^. Homozygous mutants were detected by an absence of FastRed *kni staining* during *in situ* hybridisation.

## Supporting Information

S1 File Plots of gene expression boundaries from RNAi-treated embryos of *M*. *abdita*. Summary graphs compare extracted boundary positions for wild-type (grey), and RNAi-treated embryos (coloured). Graphs are grouped by RNAi experiment as indicated by the grey bars at the top. Column headings indicate the transcript that is being displayed: *hb* (yellow), *Kr* (green), *gt* (blue), *kni* (red), and *cad* (cyan). Horizontal axes indicate % A–P position (where 0% is the anterior pole); vertical axes represent relative mRNA concentration in arbitrary units. Time flows downwards: C11–13, cleavage cycles 11–13; C14A is further subdivided into time classes T1–8 [56].
(PDF)

S2 File Plots of gene expression boundaries from RNAi-treated embryos of *D*. *melanogaster*. Summary graphs compare extracted boundary positions for wild-type (grey), and RNAi-treated embryos (coloured). Graphs are grouped by RNAi experiment as indicated by the grey bars at the top. Column headings indicate the transcript that is being displayed: *Kr* (green), and *kni* (red). Horizontal axes indicate % A–P position (where 0% is the anterior pole); vertical axes represent relative mRNA concentration in arbitrary units. Time flows downwards: C11–13, cleavage cycles 11–13; C14A is further subdivided into time classes T1–8 [12].
(PDF)

S1 Fig *otd* expression in wild-type and *bcd* RNAi knock-down embryos of *M*. *abdita*. All embryos are at time class T4. Embryo images show lateral views: anterior is to the left, dorsal is up.
(TIF)

S2 Fig *Kr* expression in wild-type and *hb* RNAi knock-down embryos of *M*. *abdita*. All embryos are at time class T4. Embryo images show lateral views: anterior is to the left, dorsal is up.
(TIF)

S3 Fig *kni* expression in wild-type and *hb* RNAi knock-down embryos of *D*. *melanogaster*. All embryos are at time class T4. Embryo images show lateral views: anterior is to the left, dorsal is up.
(TIF)
