# Supplementary material for: Maternal Co-ordinate Gene Regulation and Axis Polarity in the Scuttle Fly Megaselia abdita
Source: PLoS Genet. 2015 Mar 10;11(3):e1005042. doi: 10.1371/journal.pgen.1005042 (PMC4355411; doi:10.1371/journal.pgen.1005042)

Supporting File S1.
Plots of gene expression boundaries from RNAi-treated embryos of *Megaselia abdita*

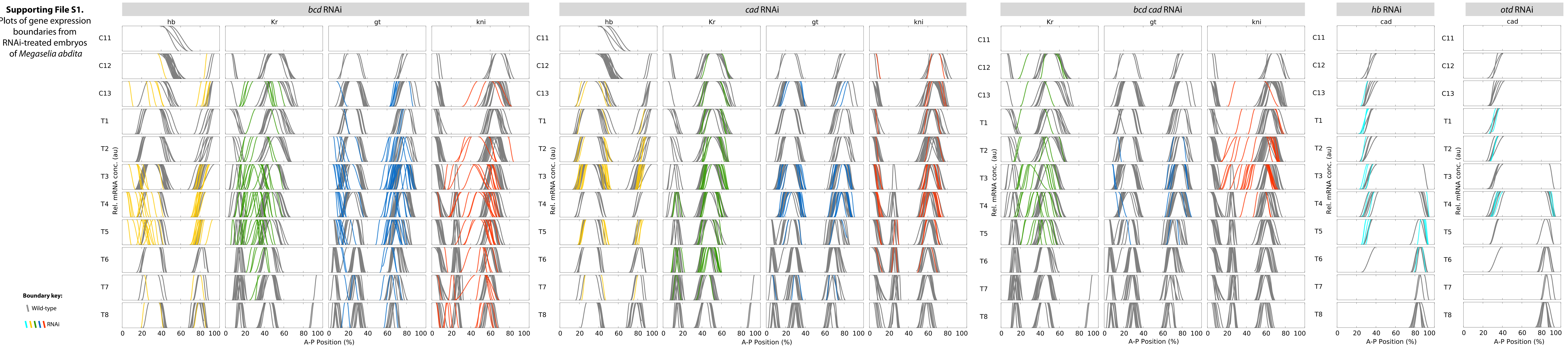

Supplement: S1 File — Summary graphs compare extracted boundary positions for wild-type (grey), and RNAi-treated embryos (coloured). Graphs are grouped by RNAi experiment as indicated by the grey bars at the top. Column headings indicate the transcript that is being displayed: hb (yellow), Kr (green), gt (blue), kni (red), and cad (cyan). Horizontal axes indicate % A–P position (where 0% is the anterior pole); vertical axes represent relative mRNA concentration in arbitrary units. Time flows downwards: C11–13, cleavage cycles 11–13; C14A is further subdivided into time classes T1–8 [56]. (PDF) [file pgen.1005042.s001.pdf]
