# Supplementary material for: Maternal Co-ordinate Gene Regulation and Axis Polarity in the Scuttle Fly Megaselia abdita
Source: PLoS Genet. 2015 Mar 10;11(3):e1005042. doi: 10.1371/journal.pgen.1005042 (PMC4355411; doi:10.1371/journal.pgen.1005042)
Supplement: S2 File — Summary graphs compare extracted boundary positions for wild-type (grey), and RNAi-treated embryos (coloured). Graphs are grouped by RNAi experiment as indicated by the grey bars at the top. Column headings indicate the transcript that is being displayed: Kr (green), and kni (red). Horizontal axes indicate % A–P position (where 0% is the anterior pole); vertical axes represent relative mRNA concentration in arbitrary units. Time flows downwards: C11–13, cleavage cycles 11–13; C14A is further subdivided into time classes T1–8 [12]. (PDF) [file pgen.1005042.s002.pdf]

Supporting File S2.
Plots of gene expression boundaries from RNAi-treated embryos of *Drosophila melanogaster*

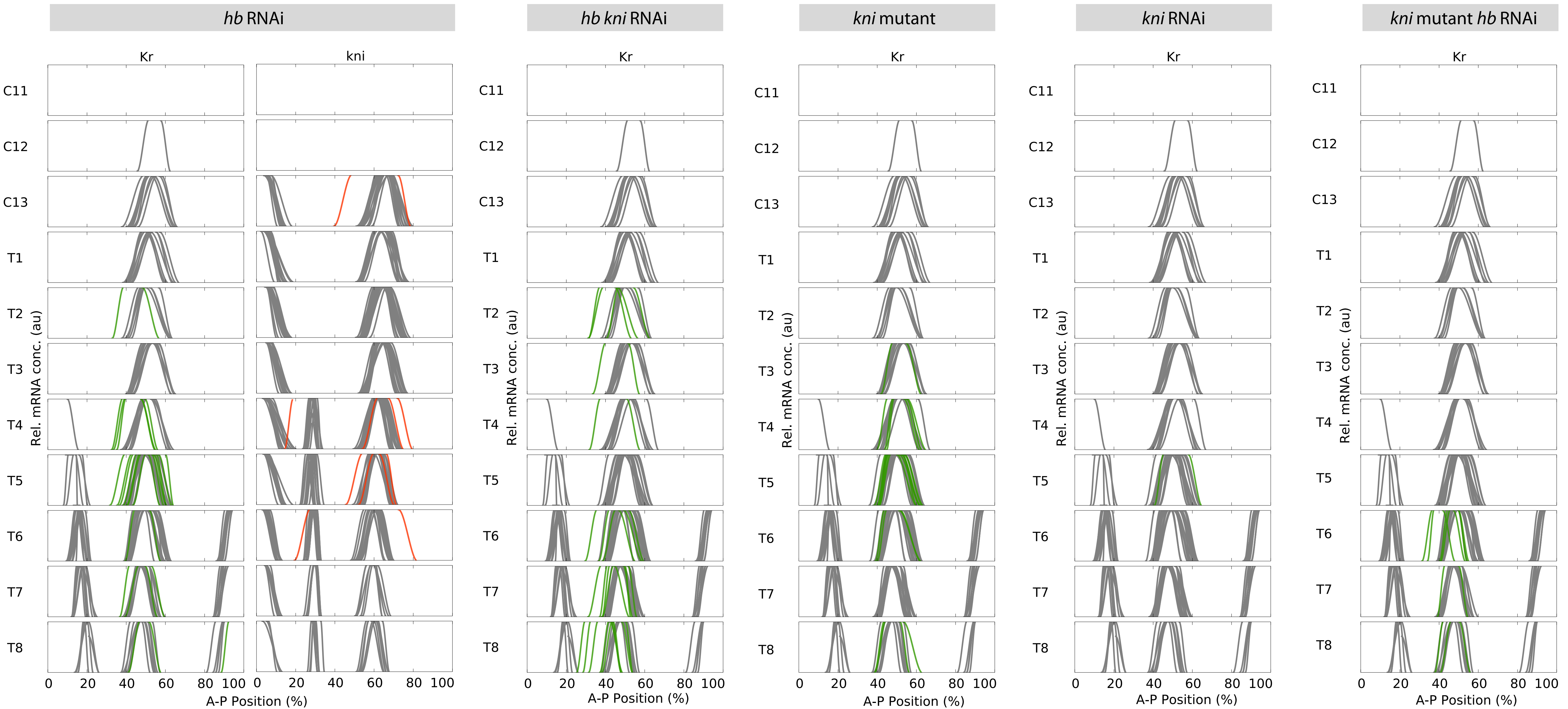

Boundary key:

Wild-type

RNAi and/or mutant
