# Supplementary figures and images for: Maternal Co-ordinate Gene Regulation and Axis Polarity in the Scuttle Fly Megaselia abdita
Source: PLoS Genet. 2015 Mar 10;11(3):e1005042. doi: 10.1371/journal.pgen.1005042 (PMC4355411; doi:10.1371/journal.pgen.1005042)

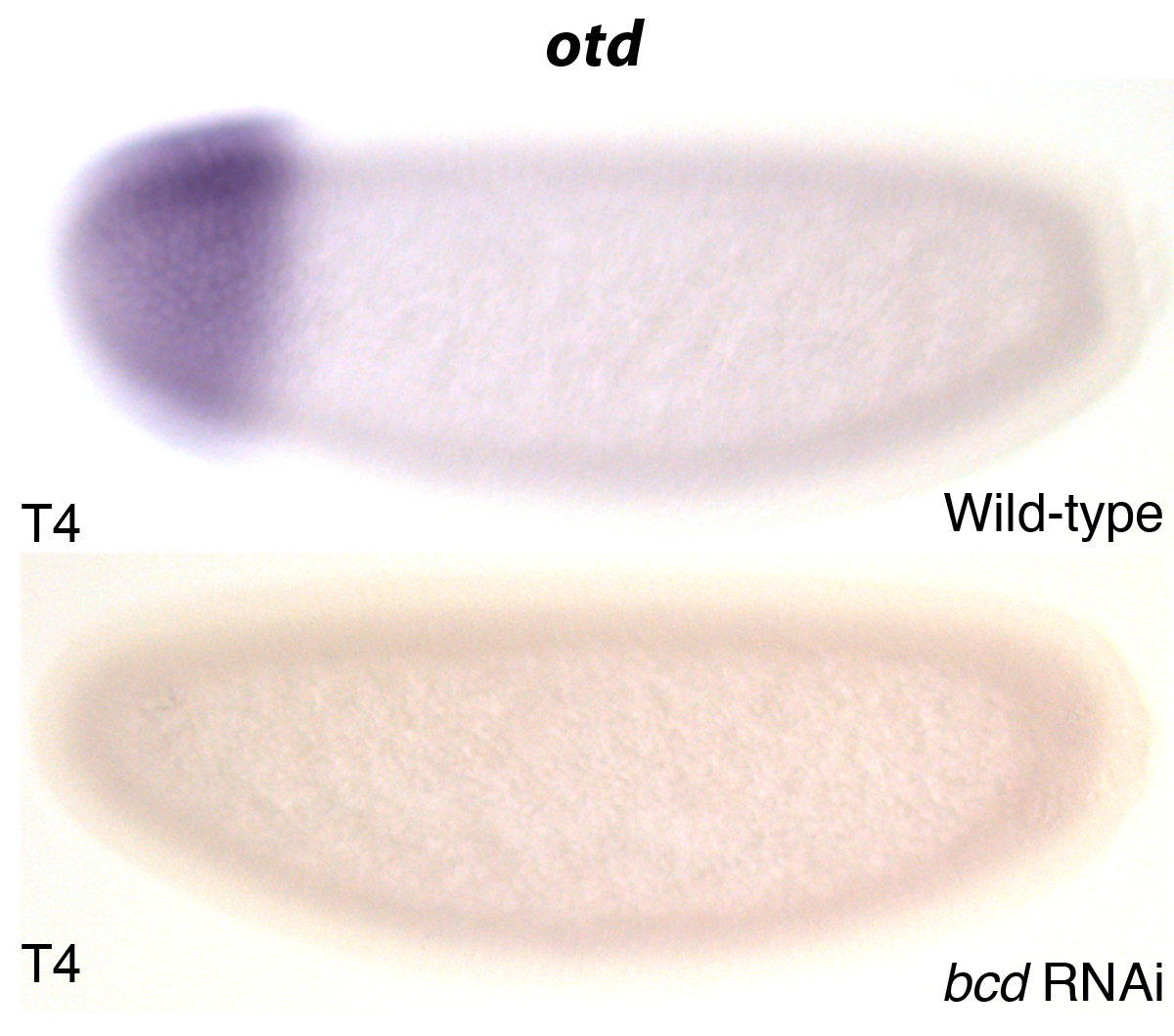

Supplement: S1 Fig — All embryos are at time class T4. Embryo images show lateral views: anterior is to the left, dorsal is up. (TIF) [file pgen.1005042.s003.tif]

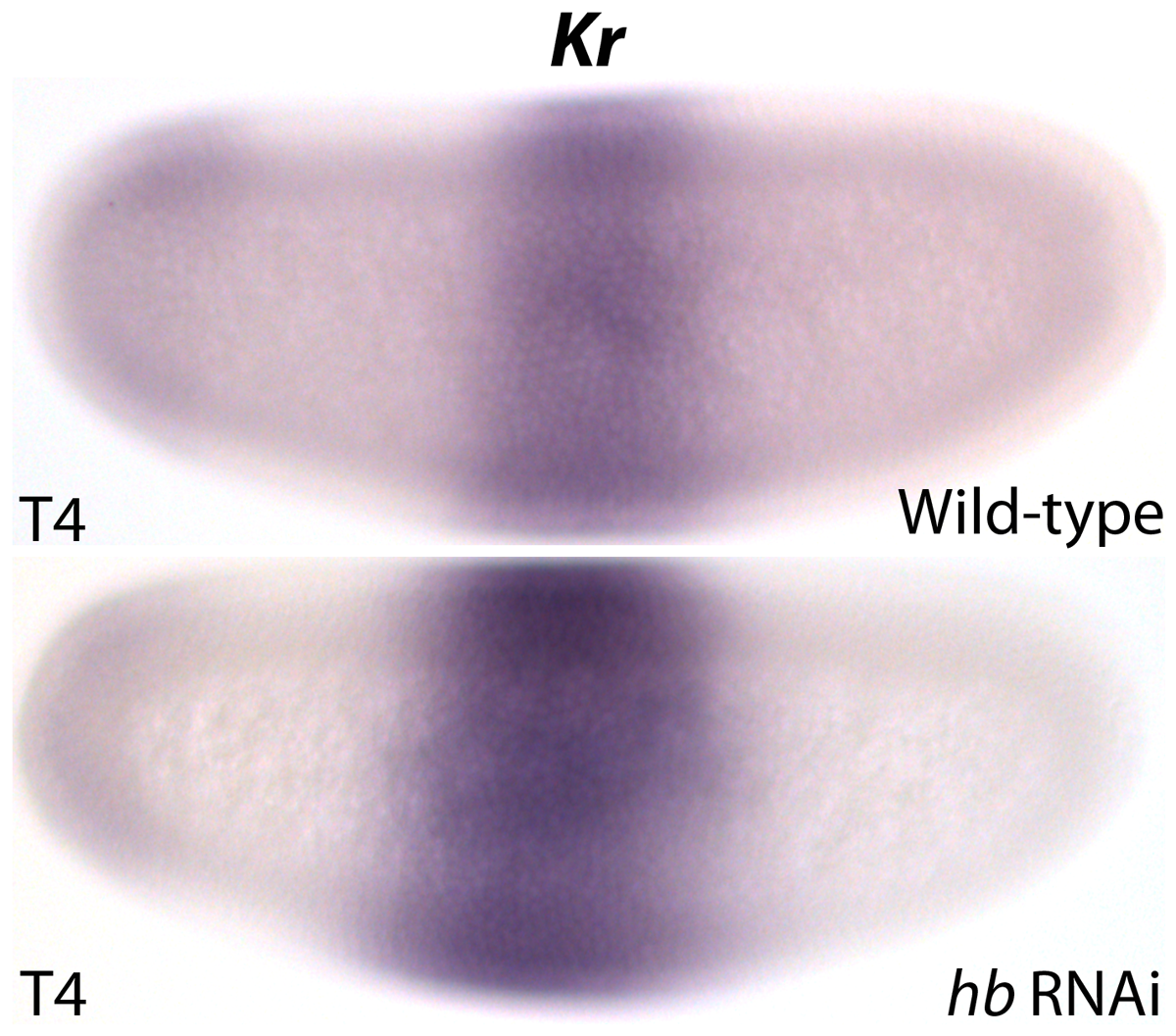

Supplement: S2 Fig — All embryos are at time class T4. Embryo images show lateral views: anterior is to the left, dorsal is up. (TIF) [file pgen.1005042.s004.tif]

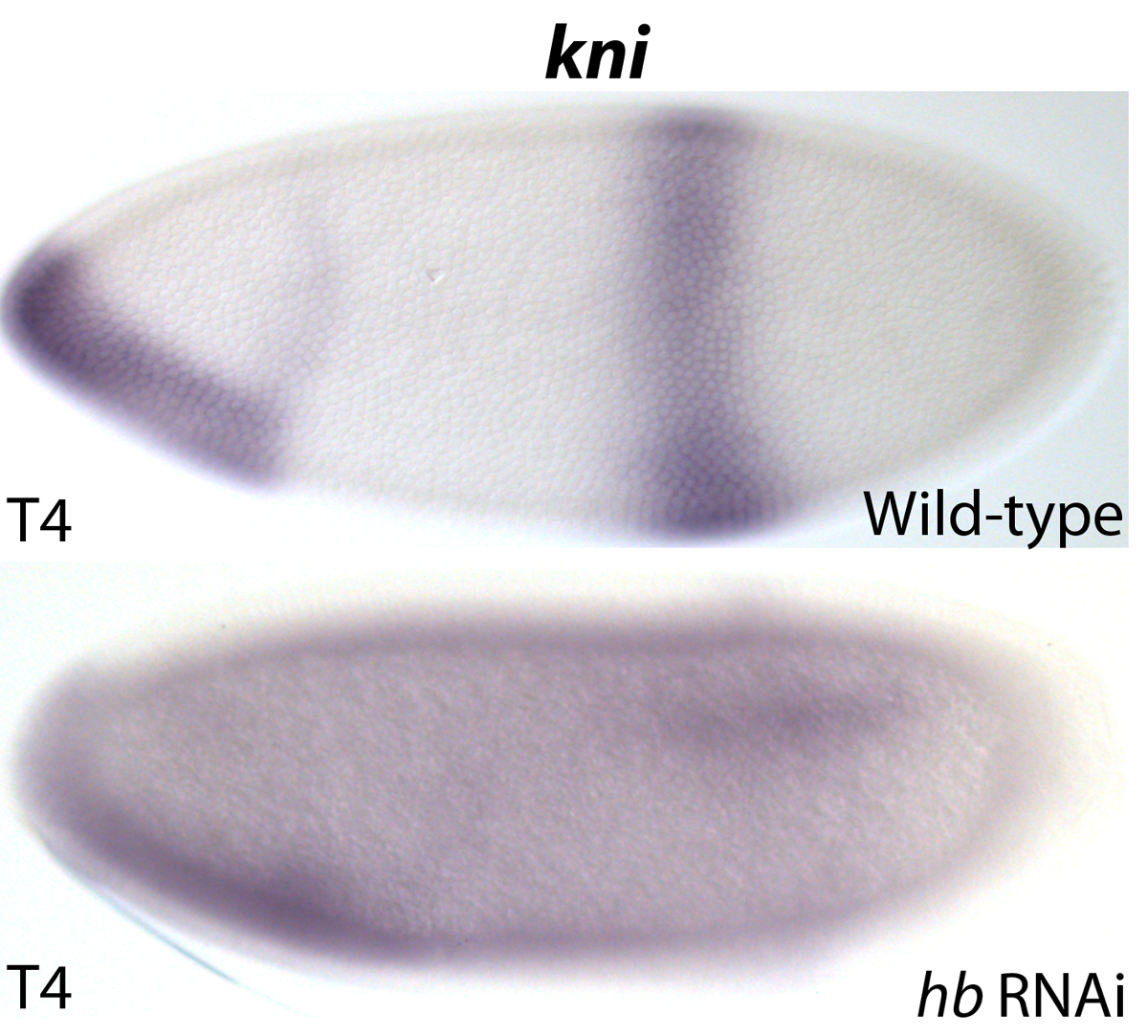

Supplement: S3 Fig — All embryos are at time class T4. Embryo images show lateral views: anterior is to the left, dorsal is up. (TIF) [file pgen.1005042.s005.tif]
